# Long-term results of high-density porous polyethylene implants in facial skeletal augmentation: An Indian perspective

**DOI:** 10.4103/0970-0358.63955

**Published:** 2010

**Authors:** Sanjeev Deshpande, Amarnath Munoli

**Affiliations:** Department of Plastic Surgery, Gokuldas Tejpal Hospital, Mumbai, India

**Keywords:** High density porous polyethylene, facial skeletal augmentation, alloplast

## Abstract

**Context:**

With the increasing emphasis on well-sculpted facial features, today there is a growing need for tools to augment the facial skeleton; either for cosmetic reasons or to re-contour deformities-congenital, post-traumatic and post-ablative. The limitations of autogenous materials has lead to evolution of numerous 'alloplasts', of which, high-density porous polyethylene (HDPE) seems to be a promising alternative.

**Aims:**

To evaluate the long term results of HDPE in facial skeletal augmentation in terms of achieving desired facial contour, patient satisfaction and complications.

**Settings:**

A tertiary care referral centre in a metropolitan set-up.

**Design:**

Case Series

**Materials and Methods:**

All patients undergoing HDPE implant insertion for facial skeletal augmentation between July 2001 and November 2009 were included in the study. A total of 70 HDPE implants were inserted in 44 patients. All procedures were performed by a single surgeon following standardized pre, intra and post-operative protocols. The results were evaluated with respect to improvement in facial contour desired and achieved, overall patient satisfaction and complications encountered.

**Results:**

The study included 44 patients with a male:female ratio of 1:1, a mean age of 25.09 years (14 to 58 years) and a mean follow-up of 45.34 months (0.5 to 100 months). HDPE implants were used to augment the nasal dorsum, maxilla, malar eminence, chin, mandibular body and angle, orbital rim and frontal region. The overall recontouring afforded by the HDPE implants was good, with most patients reporting satisfactory results. There were seven complications (10%), including three cases of deviation (4.29%), three cases of exposure (4.29%) and one case of sub-clinical infection (1.43%). None however necessitated implant removal. Nasal dorsal HDPE implants, especially those involving secondary surgery, suffered a much higher complication rate compared to other implants.

**Conclusions:**

HDPE is an alternative to autogenous grafts for facial skeletal augmentation with good long-term results and a low incidence of complications, provided there is adequate vascular soft tissue cover.

## INTRODUCTION

In an increasingly image-conscious world, where 'looking right' can make all the difference[1], the desire to possess 'perfect facial features' is universal. Not surprisingly, today's Plastic Surgeon is frequently confronted with requests for alterations in facial profile for a host of reasons[2]: congenital, post-traumatic, post-ablative deformities or purely cosmetic considerations. A fair percentage of these requests pertain to recontouring the skeletal features of the face –cheek bones, nose, chin and forehead.

Facial skeletal contouring has come a long way from its early days when Tessier[3] showed the world that radical alterations in the facial skeleton were possible through osteotomies and grafting. Traditionally regarded as the 'gold standard'[4] for facial reconstruction, autogenous bone grafts have several disadvantages[5,6] including donor site morbidity, resorption, difficulties in carving and additional operative time.

This lead to a search for the ideal alloplastic material[7,8] to replace or augment the skeleton – chemically inert, biocompatible, non-allergenic, non-carcinogenic, sterilizable, easy to handle, stable, radio-opaque, cost-effective, permitting tissue in-growth. Although no single alloplast fulfilling all these criteria has been discovered till date, high density porous polyethylene (HDPE) - a large pore, biocompatible, synthetic material—appears to be suitable for maxillofacial skeletal reconstruction.

Solid Polyethylene was first used as a substitute for bone or cartilage in humans in the 1940s[9] with favourable results. Porous high-density polyethylene was developed in the early 1970s and has been in use since. We present here the long term results of HDPE implants in maxillofacial skeletal augmentation, performed by a single surgeon in an Indian setting.

## MATERIAL AND METHODS

We reviewed 44 successive patients who underwent facial skeletal augmentation with HDPE implants for several indications from July 2001 to November 2009. The sample included 22 male and 22 female subjects with age at the time of surgery ranging from 14 to 58 years (mean age = 25.09 years). The various indications for implant placement are presented in Table 1.

**Table 1:** Indications of HDPE implant insertion

*Sr.No*.		*Indications*	*Number of patients*
1	Congenital	Cleft Lip and Palate(with 1 case of Tessier cleft type 0	6
		Hemifacial microsomia	2
2	Post-traumatic		4
3	Post-ablation Reconstruction	Neurofibroma	1
		Nose reconstruction	1
		Post-radiotherapy	1
		Zygomatic tumour	1
4	Aesthetic		28
Total			44

A total of 70 HDPE implants were used for augmentation of nasal dorsum (28), maxilla (22), malar eminence (6), chin (5), mandibular body and angle (4), orbital rim (3) and frontal region (2). During the course of post-operative follow-up, three patients wished for augmentation of other regions of their facial profile and thus they were operated twice.

A detailed written informed consent was obtained from all patients after explaining the nature, merits and possible adverse effects of the implants. All the procedures were performed under General Anaesthesia and preceded by the infiltration of diluted epinephrine solution (1 in 2 lacs dilution). A standardized surgical procedure was followed for each implant site in terms of approach, extent of sub-periosteal dissection, implant handling/carving, implant fixation, wound closure and antibiotic cover. The exact details of the procedure of implant insertion at various sites are well documented.[8,12‐16]

In post-traumatic cases, previous scars were used for approaching the facial skeleton wherever possible in combination with revision of scars (Case5).

In most cases, we used pre-fabricated, contoured implants with final moulding and shaping done intra-operatively after assessing the augmentation desired and obtained after placing the implant in its sub-periosteal pocket. Any necessary carving or shaping was done using a fresh scalpel blade no.21.

Patients received one dose of antibiotic pre-operatively, which was continued for seven days post-operatively. The average hospital stay was five days.

The results were analyzed by comparing pre and post-operative photographic documentation (standard six views) as well as assessment during follow-up examinations. This included patients' overall satisfaction with respect to desired and perceived cosmetic benefit and functional outcome. Patients were instructed to provide their insights regarding the success/failure of the augmentation by grading the final result as—extremely pleasing, pleasing, satisfactory, not satisfactory and disappointing. Any complications or complaints related to the implant placement were duly noted and documented.

## RESULTS

**Figure 1:**
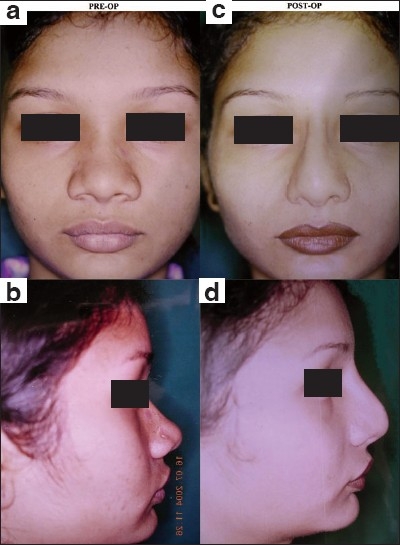
Nasal Dorsal Augmentation with HDPE implant: (a and b) Pre-operative frontal view and profile showing depressed nasal dorsum and wide alar base; (c and d) Post-operative frontal view and profile demonstrating effective augmentation of the nasal dorsum by HDPE implant with alar base narrowing

**Figure 2:**
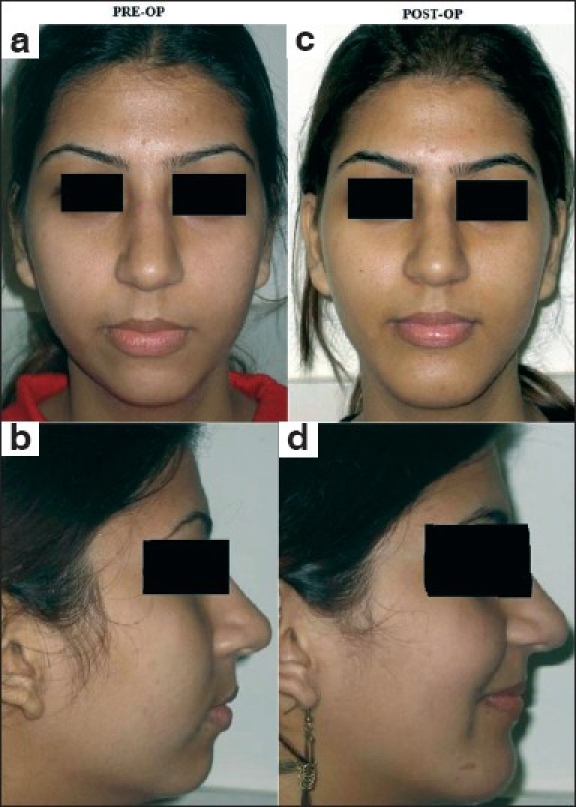
Chin augmentation with pre-formed HDPE chin implant in a young female with microgenia: (a and b) Pre-operative frontal view & profile showing unnatural appearance due to the retruded chin; (c and d ) Post-operative pictures illustrating the marked improvement in facial profile following insertion of a HDPE chin implant through intra-oral approach

**Figure 3:**
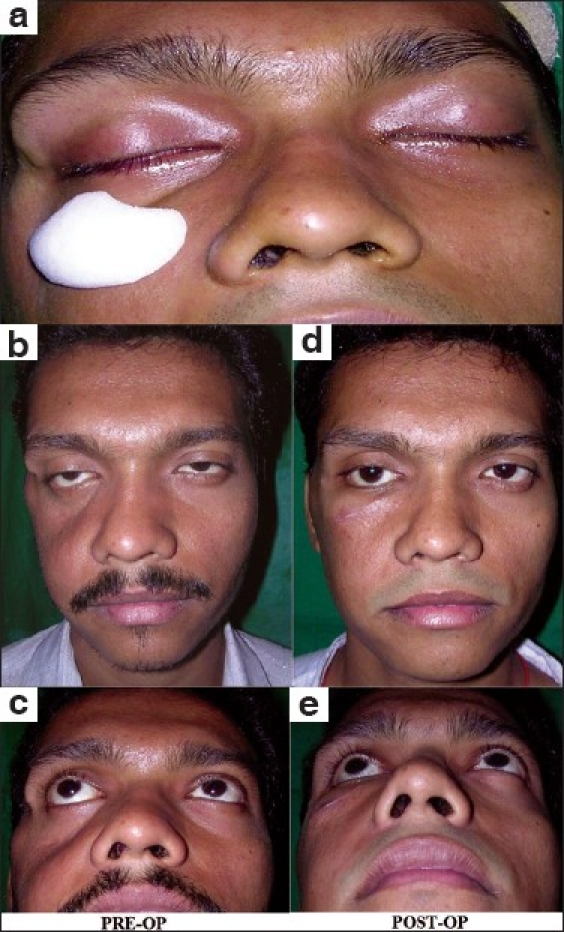
Malar augmentation with HDPE implant in a case of old untreated fracture zygoma with Right malar depression: (a) Intra-operative photograph showing the approximate position for implant placement and pocket dissection; (b and c) Pre-operative frontal & worm's eye views demonstrating marked hollowing in the right malar region. Also notice the drooping of the right lower eyelid and mild enophthalmos; the patient had no visual complaints; (d and e) Post-operative pictures illustrate the excellent contour provided by the malar implant; the salutary effect on the lower eyelid drooping is remarkable

**Figure 4:**
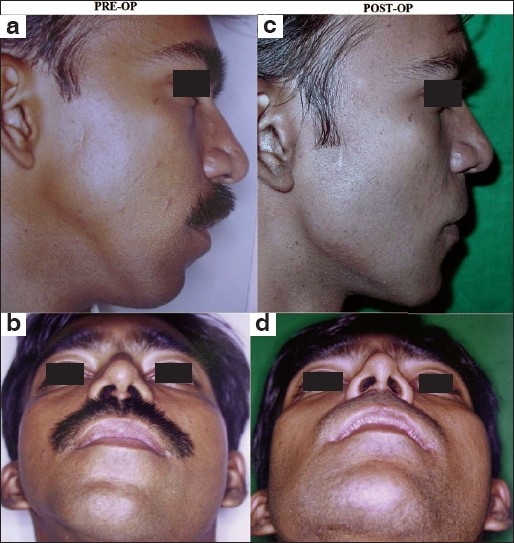
Augmentation of the mandibular angle and body with pre-formed HDPE implants: (a and b) Pre-surgical appearance of a young male subject with right hemifacial microsomia mainly involving the lower jaw. He had a fairly decent occlusion and wished only for cosmetic improvement of the mandibular deformity; (c and d) Post-operative profile and worm's eye views showing creation of a well-defined lower jaw line on the right side with restoration of facial symmetry.

**Figure 5:**
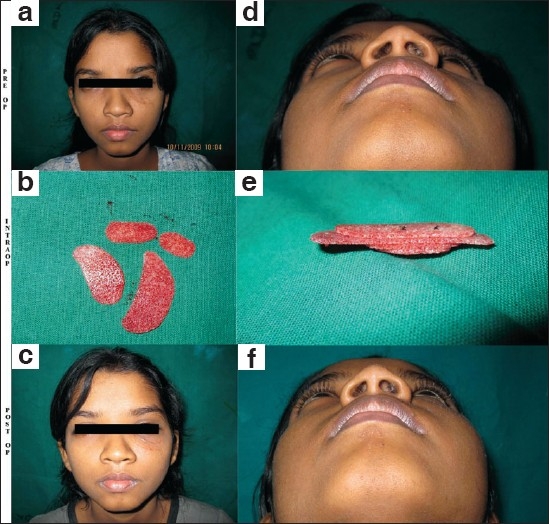
Recreation of infra-orbital rim in a young girl with old fracture of left zygoma involving bone loss, with depressed left infra-orbital region: (a and d) Pre-operative frontal and worm's eye views showing visible depression and scars of previous surgery in the left infra-orbital region. There were no visual complaints, enophthalmos or hypesthesia; (b and e) Showing the HDPE sheet cut into appropriate shapes according to the defect and then stacked and fixed together with non-absorbable suture; (c and f) Post-operative pictures demonstrating suitable contour correction with the "customised" implant.

A total of 70 HDPE implants were inserted in 44 patients between July 2001 and November 2009.These included implants for the nasal dorsum (28) [Figure 1], maxilla (22), malar eminence (6) [Figure 3], chin (5) [Figure 2], mandibular body & angle (4) [Figure 4], orbital rim (3) [Figure 5] and frontal region (2). The series included 22 male and 22 female subjects with ages ranging from 14 years to 58 years, the mean age being 25.09 years. The follow-up period ranged from 15 days to 100 months with an average of 46.34 months.

**Figure 6:**
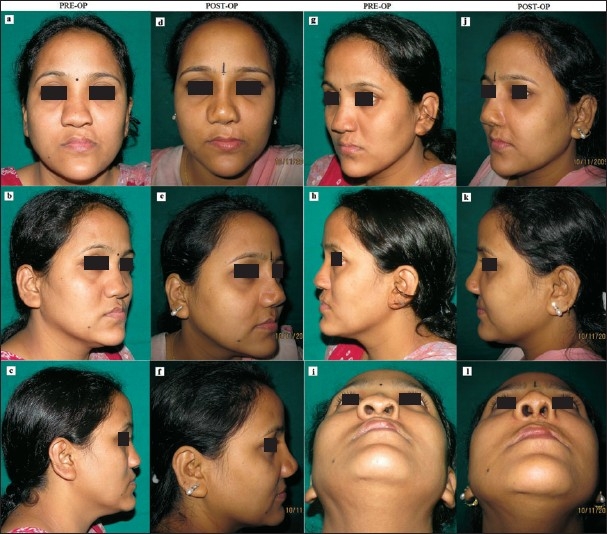
Bilateral paranasal, pre-maxillary and nasal dorsal augmentation with HDPE implants in an operated case of Left sided Cleft lip and Palate: (a, b, c, g, h and i) Pre-operative 6 standard views showing the stigmata of cleft lip and palate-- depressed nasal dorsum, drooping tip & bilateral maxillary hypoplasia; (d, e, f, j, k and l) Post-operative pictures show improved nasaldorsum and tip projection with adequate fullness in the pre-maxillary and paranasal regions

A comparison of preoperative and postoperative photographs (immediate & long term) revealed that HDPE implants provided effective and stable augmentation of the desired facial contour while maintaining a natural appearance and feel, as is quite evident from the photographs [Figures 1‐6]. During follow-up analysis, almost all patients expressed satisfaction with the aesthetic enhancement afforded by the implant as compared to their preoperative appearance.

Overall, we had a patient grading as follows: 28:extremely pleasing, 13:pleasing, 2:satisfactory and 1: not satisfactory. Thus, 41 patients (93.18%) were pleased with their results. Only three patients who had undergone nasal dorsal augmentation complained of unnaturally hard feel of the nose; however, they were pleased with the final result and did not demand removal of the implant.

None of the patients reported abnormal movement, displacement or migration of their implants or any evidence of discomfort, paresthesia at the implant site, during the follow-up period.

## COMPLICATIONS

In the present series of 70 implants, we had seven complications (10%)—three cases of deviation of implant (4.29%), one case of sub-clinical infection (1.43%) and three cases of implant exposure (4.29%). These complications involved six nasal dorsal implants and one paranasal implant.

Of the three deviated nasal dorsal implants, only one occurred in the late post-operative period (8 months post-operatively) and needed additional fixation with a mini-screw; the remaining two occurred early (within 15 days post-operatively) and responded to manipulation followed by splintage for one week—there was no recurrence. There was one case of sub-clinical implant infection 10 months post-operatively, presenting as erythema & warmth at the nasal tip, which resolved completely after a course of oral antibiotics and did not recur.

We had three cases of implant exposure in two patients—in the first, a nasal dorsal implant was seen through the nostril associated with signs of infection, 18 months after implant insertion; in the second case, a nasal dorsal implant was exposed through the nostril 10 months after insertion associated with inflammatory signs. In both cases we found that trimming the exposed part of the implant sufficiently to obtain a tension free closure of the overlying tissues along with a week of intravenous antibiotics was adequate and we have not had any further problems with either patient. In case of the second patient, a case of Tessier cleft type 0, we found a small area of exposed paranasal implant in the alveolus with no adverse signs or symptoms for three months, post-operatively. We have monitored this patient regularly for any signs of inflammation or further breakdown over the past 12 months and continue to do so.

We did not encounter breakdown, fracture or gross displacement of the implant in any of our patients.

## DISCUSSION

Augmentation of various aspects of the facial skeleton for aesthetic and reconstructive reasons can be achieved by the traditional tools of orthognathic surgery, namely osteotomies, bone graft and prolonged orthodontic treatment.[4,10] However, for a patient with no functional problems (e.g. malocclusion) seeking a quick, relatively minor cosmetic procedure, these would seem unacceptably complex and costly. Also, increasingly, patients turn up at consulting rooms who have already undergone almost the entire range of orthognathic procedures for facial skeletal correction—these include patients with congenital anomalies, facial bone fractures and post-resection maxillofacial reconstruction. This group of patients not only desires simple, specific solutions, but may also have inadequate quantity/quality of bone stock for grafting & osteotomies. We also cannot overlook the significant disadvantages of bone grafts.[5,6]

Facial skeletal augmentation with alloplastic materials is a well-established technique with a low incidence of complications.[7,8,11,12] Our quest for the ideal alloplast has yielded innumerable materials in the past several decades, some of which (like Proplast) have already been abandoned.

Polyethylene has been used as an implant material for more than 60 years. Solid Polyethylene was first used as a substitute for bone or cartilage in humans in 1947[9] with favourable results. HDPE became commercially available as MEDPOR (Porex Surgical, College Park, GA) in 1985. BIOPORE has been available in the Indian market since 2006 (www.biopore.in).

HDPE is a medical grade, inert, radiolucent, pure, linear polyethylene, sintered to form a framework of interconnecting pores. Physically, it is strong with good tensile strength, resistant to stress and fatigue, biocompatible, with minimalsurrounding soft tissue reaction and extremely stable in the long term; there are reports of more than 30 years of follow-up.[9]

In spite of its strength, HDPE is technically easy to work with—it is thermoplastic, can be carved with sharp instruments, sheets can be easily cut with scissors, sutures can be passed through it and it is readily stabilised with screws. It can be tailored to a specific patient's needs based on stereolithographic reconstruction from a 3-dimensional CT scan.

The large & stable pores of HDPE promote a rapid bony and fibrous ingrowth into the implant[13,17] which, in turn: minimizes capsule formation, anchors the implant, maintains the local host immune response.

In our study we found that there was bleeding from the cut surface of the implant during revision surgery; the same finding has been reported by others.[14,15,18]

In our series, we found that HDPE implants are universally easy to shape, carve & contour, regardless of the make of the implant or the nature of the defect. Moreover, they are highly adaptable and lend themselves to restructuring and remodelling, for example, we used carefully shaped & stacked sheets of HDPE for augmenting the nasal dorsum (1 case), infra-orbital rim (2 cases) with excellent results [as seen in Case 5].

Although we performed all augmentations under General Anesthesia, the absence of a donor site for graft harvest would make the use of sedation and regional blocks an extremely viable option—with careful patient selection, one may even consider the possibility of inserting implants as a day-care procedure. Of the 44 patients reviewed, 15 were undergoing revisional surgery, of these, five had been operated once before; the remaining 10 had undergone two or more previous surgeries. We did not encounter any significant difficulty in the insertion of implants in this sub-group despite the fibrosis at the approach sites & the scarred beds. In fact, in one patient, we successfully inserted a HDPE implant for nasal dorsal augmentation under an area of previous grafted skin (STSG), the patient having undergone excision of neurofibromatosis over her face followed by STSG.

Overall, patients were satisfied with the aesthetic results of the alloplastic augmentation, as evaluated during post-operative follow-ups, with 93.18% patients saying they are extremely pleased or pleased with their results. A review of literature reveals numerous experimental, animal, histologic and clinical studies confirming the safety and efficacy of HDPE implants in aesthetic and reconstructive craniofacial surgery.[13,14,19,20] We believe that ours is the first comprehensive review of the long term results of HDPE implants for facial skeletal augmentation to come out of India.

We had a total of seven complications (10%) among 70 implants inserted with three instances of implant exposure (4.29%), however significantly, these exposed implants could be trimmed and covered by secondary suturing and none necessitated implant removal. These percentages compare favourably with published foreign series[13‐15,20,21] reporting results of HDPE implants where the complication rate & exposure rate range from 0-29%[22] and 0-9.7%.[22]

In our experience, six of the seven reported complications and two of the three exposures involved nasal dorsal implants (28 in number). Thus, the nasal dorsal implants suffer a higher complication and exposure rate (21.43% & 7.14%), a finding which is again comparable with several series from the West.[13,21] On the other hand, only one of the seven complications and one of the three exposures involved the '*non-nasal dorsal implants*' (42 in all). Thus, excluding HDPE implants for nasal dorsal augmentation, the remaining implant sites suffered a complication rate and exposure rate of only 2.38% each.

Of the 15 patients undergoing revision surgery, 12 involved nasal dorsal augmentation; i.e. 43% (12/28) nasal dorsal HDPE implants were inserted in previously operated noses. This may account for the higher complication rate in this group of patients in our series. Both the exposed nasal implants involved secondary rhinoplasty patients who had already undergone nasal surgery in the past, leading to thinned out, scarred and fibrotic tissue in the nasal vestibule and implant exposure through the intra-nasal incision site. We believe that adverse results in this group of patients can be avoided by two simple steps—carving an implant of a size 0.5cm shorter than the anticipated requirement and carefully trimming the tip of the implant to a smooth contour, since this is the part that invariably gets exposed due to pressure necrosis of the overlying tissues.

## CONCLUSION

Aesthetic facial skeletal augmentation with HDPE implants is an easy, safe and effective procedure with no donor site morbidity, excellent and stable contour enhancement and minimal long-term complication rate. Considering the higher complication rate, it would be prudent to exercise caution in the use of nasal dorsal HDPE implants in cases of secondary surgery with fibrotic tissues. It is vital to counsel patients and establish realistic and precise aesthetic goals preoperatively.
